# Distinct and Histone-Specific Modifications Mediate Positive versus Negative Transcriptional Regulation of TSHα Promoter

**DOI:** 10.1371/journal.pone.0009853

**Published:** 2010-03-24

**Authors:** Dongqing Wang, Xianmin Xia, Roy E. Weiss, Samuel Refetoff, Paul M. Yen

**Affiliations:** 1 Endocrinology Division, Department of Medicine, Johns Hopkins University School of Medicine, Baltimore, Maryland, United States of America; 2 Laboratory of Hormonal Regulation, Cardiovascular and Metabolic Disease Program, Duke-National University of Singapore Graduate Medical School, Singapore, Singapore; 3 DJ Biotech, Changzhou, China; 4 Departments of Medicine and Pediatrics, University of Chicago, Chicago, Illinois, United States of America; Texas A&M University, United States of America

## Abstract

**Background:**

Hormonally-regulated histone modifications that govern positive versus negative transcription of target genes are poorly characterized despite their importance for normal and pathological endocrine function. There have been only a few studies examining chromatin modifications on target gene promoters by nuclear hormone receptors. Moreover, these studies have focused on positively-regulated target genes.

TSHα, a heterodimer partner for thyrotropin (TSH), is secreted by the pituitary gland. T_3_ negatively regulates TSHα gene expression via thyroid hormone receptors (TRs) which belong to the nuclear hormone receptor superfamily, whereas thyrotropin releasing hormone (TRH) positively regulates via the TRH receptor, a G protein-coupled receptor.

**Methodology/Principal Findings:**

We studied regulation of the TSHα gene by cAMP and T_3_ using chromatin immunoprecipitation (ChIP) assays in stably-transfected rat pituitary cells containing the human TSHα promoter. Interestingly, cAMP selectively increased histone H4 acetylation whereas, as previously reported, T_3_ induced histone H3 acetylation. In particular, cAMP increased H4K5 and H4K8 acetylation and decreased H4K20 trimethylation, modifications associated with transcriptional activation. T_3_ increased H3K9 and H3K18 acetylation and H3K4 trimethylation; however, it also decreased H3K27 acetylation and increased H3K27 trimethylation which are associated with transcriptional repression. Of note, cAMP recruited pCREB, CBP/p300, and PCAF to the promoter whereas T_3_ caused dissociation of NCoR/SMRT and HDAC3. Overexpression of a dominant negative mutant thyroid hormone receptor (TR) from a patient with resistance to thyroid hormone (RTH) led to less T_3_-dependent negative regulation and partially blocked histone H3 modifications of the TSHα promoter.

**Conclusions/Significance:**

Our findings show that non-overlapping and specific histone modifications determine positive versus negative transcriptional regulation, and integrate opposing hormonal and intracellular signals at the TSHα promoter. A mutant TR from a patient with RTH exerted dominant negative activity by blocking the histone modifications induced by T_3_ on the TSHα promoter and likely contributes to the inappropriate TSH production observed in RTH.

## Introduction

Thyroid hormone receptors (TRs) are members of the nuclear hormone receptor superfamily that are responsible for many of the physiological and cellular effects by thyroid hormones. Moreover, in response to triiodothyronine (T_3_), TRs mediate positive and negative transcription of target genes [1], [2]. In positively-regulated target genes, unliganded TRs bind to thyroid hormone response elements (TREs) in the promoters of target genes. They also associate with corepressors such as nuclear receptor corepressor (NCoR) or silencing mediator for retinoic and thyroid hormone receptors (SMRT) in corepressor complexes also containing transducin β-like protein 1 (TBL1) and histone deacetylase 3 (HDAC3). These complexes cause histone deacetylation of the promoter region near the TRE, heterochromatin formation, and transcriptional repression [3]–. When T_3_ is present, corepressor complexes dissociate from liganded TRs whereas coactivator complexes that contain steroid receptor coactivator (SRCs), CBP, and P/CAF bind to TRs. These coactivator complexes contain histone acetyltransferase (HAT) activity and induce histone acetylation near the TREs of target genes [6]–[8]. Additionally, histone demethylation also occurs on some nuclear receptor target genes, particularly H3K4 and H3K9 [9]–[12]. ATP-dependent chromatin remodeling complexes similar to yeast SWI/SNF which contain the ATPase subunit, Brahma-related gene (BRG1) also are recruited to the promoter [13], [14] and likely facilitate chromatin renodeling necessary for HAT activity by SRC complexes and demethylases. Another major complex, Mediator, interacts with liganded TRs and recruits RNA polymerase II (RNA pol II) to the transcriptional start site [6], [15], [16].

Despite evidence that the majority of target genes appear to be negatively-regulated by T_3_ [17], the mechanism(s) for negative transcriptional regulation is not well understood. For several negatively-regulated genes, NCoR and SMRT increase basal transcription of target genes in the absence of T_3_ [18]–[21]. Coactivators also may be involved in T_3_-dependent negative regulation [22]. For the TSHα gene, we recently showed that a corepressor complex containing NcoR/SMRT, TBL1, and HDAC3 binds to the promoter of the TSHα gene and mediates basal transcription in the absence of T_3_ [23]. However, when T_3_ is present, this corepressor complex is released from TR, which, in turn, leads to increased histone acetylation and negative regulation of the TSHα gene expression [23]. These unexpected results demonstrate that histone acetylation per se does not universally lead to transcriptional activation.

Previous studies have shown that the hypothalamic tripeptide, thyrotropin releasing hormone (TRH), stimulates adenyl cyclase activity and induces cAMP production leading to increased TSHα gene expression [24]. The TSHα gene promoter has two cAMP response elements (CREs) [19] and dibutyryl cAMP also can activate TSHα gene transcription [22]. We previously generated a permanently-transfected rat pituitary cell line (α-23) that contains luciferase cDNA under the control of the human TSHα promoter (−846 to +1) [23]. This portion of the promoter contains the two CREs and is negatively regulated by T_3_. In this manuscript, we examine the transcriptional mechanisms and histone modifications of cAMP and T_3_ on the TSHα gene promoter. Our findings show that although common general mechanisms may be utilized for positive and negative regulation, in a given target gene such as TSHα, different co-factors as well as histone modifications at distinct sites, may determine positive versus negative regulation of transcription. Moreover, the particular constellation of histone modifications at the promoter may enable integration of opposing hormonal and intracellular signals at the target gene promoter. Last, we show that in the genetic syndrome of RTH, disruption of T_3_-dependent histone modifications by a dominant negative mutant TR may lead to inappropriate TSHα secretion.

## Results

**Figure 1:**
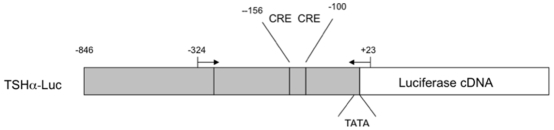
Diagram of TSHα promoter and location of primers in ChIP assays. Shown is the promoter region and luciferase cDNA. The upstream primer started at −324 bp and the downstream primer started at +23 bp from transcriptional start site of luciferase gene. Primers denoted by arrows. CRE-cyclic AMP response element; TATA-TATA box.

We previously generated a permanently-transfected pituitary cell line (α−23 cells) which incorporated a human TSHα promoter/luciferase cDNA construct (Figure 1) that was negatively-regulated by T_3_ [23]. This line was generated from somatrope/lactotrope GH3 cells since there are no readily-cultured pituitary cell lines which express TSHα that can be negatively-regulated by T_3_. Additionally, no significant amounts of endogenous TSHα mRNA could not be detected by quantitative RT-PCR and microarray analyses (data not shown).

**Figure 2:**
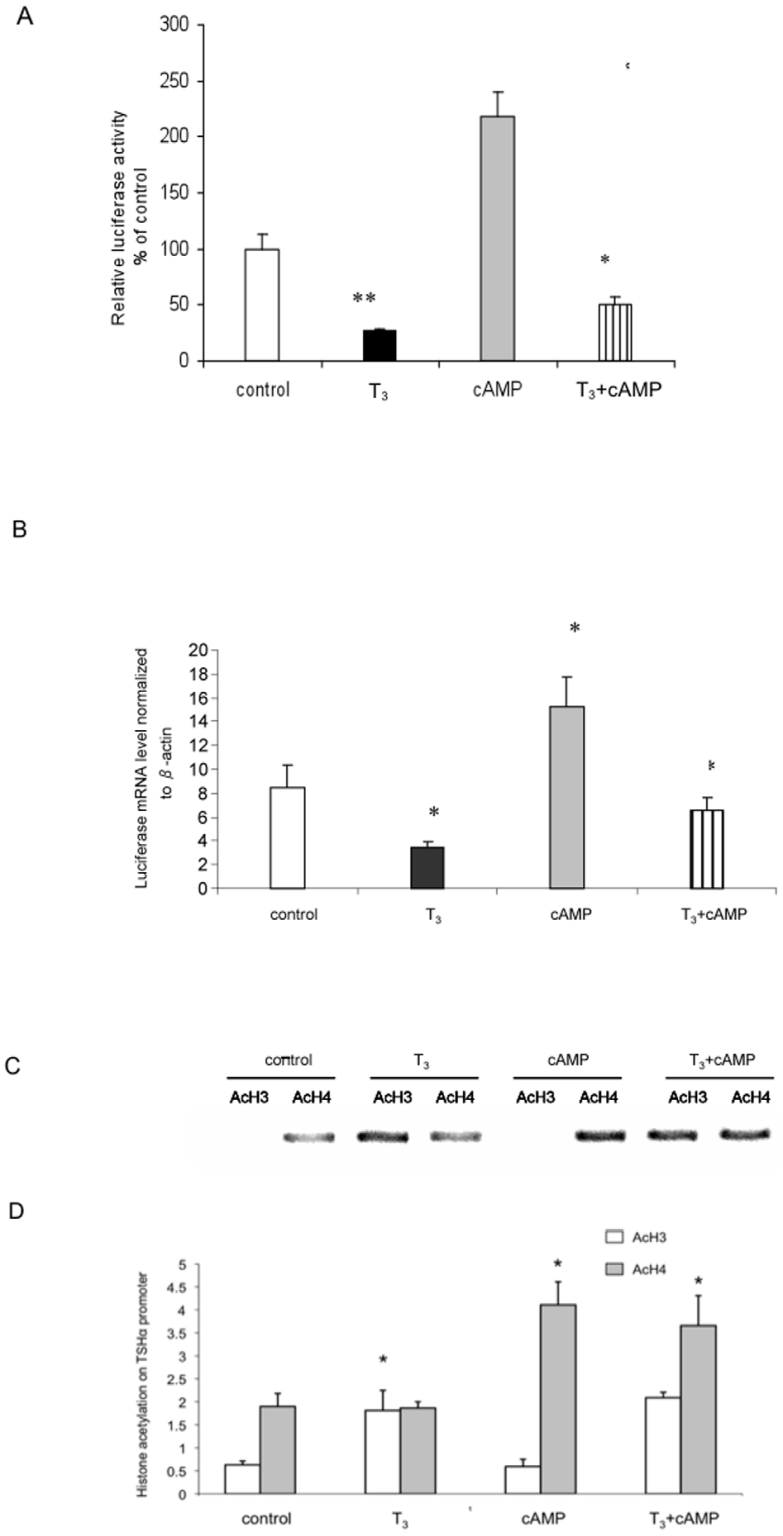
T_3_ and cAMP regulation of TSHα promoter activity and histone acetylation. A) T_3_ and cAMP regulation of TSHα promoter activity. α-23 cells were treated 24 hours with no hormone/cAMP (control), 0.1 µM T_3_, 1 mM dbcAMP, or both. The cell lysates were prepared and luciferase activities were measured as described in Materials and Methods. B) Acute T_3_ and cAMP regulation of TSHα promoter activity. Luciferase mRNA expression was analyzed by RT-PCR in cells treated for one hour with no hormone/cAMP (control), 0.1 µM T_3_, 1 mM dbcAMP, or both. β-actin gene expression was used for normalization. Shown in A and B are the mean of triplicate samples ± SD. normalized to control as 100%. Similar findings were found in two other experiments. **, p<0.05; *, p<0.01 difference from control using ANOVA analyses. C) T_3_ and cAMP regulation of histone H3 and H4 acetylation. ChIP assay was performed as described in Materials and Methods using antibodies against acetylated H3 or H4. Similar findings were observed in two other experiments. Note: “- ” indicates no hormone or cAMP treatment. D) Quantitative RT-PCR analyses of PCR products from three separate ChIP experiments. The statistical analysis was performed utilizing ANOVA. Values are expressed as the mean ± SD. *, *P*<0.05 difference from control.

We examined the transcriptional activity of human TSHα promoter in α−23 cells by measuring luciferase activity and mRNA expression by quantitative RT-PCR (Figure 2A, B). Negative regulation of transcription by T_3_ and positive regulation by cAMP were seen as early as one hour. Negative regulation also was observed in cells treated with both T_3_ and cAMP; however, the level of transcription was higher than T_3_ alone. The mean fold-change in transcription observed in cells treated with cAMP *vs*. cAMP + T_3_, was similar to control *vs*, T_3_ alone (3.6 +/− 0.5 s.d. *vs.* 3.9 +/− 0.4 s.d., respectively).

We next used ChIP assay to examine the acetylation of histones H3 (H3) and H4 (H4) under all four conditions (Figure 2C,D). The integrated human TSHα promoter region could be distinguished from the endogenous rat TSHα promoter due to specific amplification using a downstream luciferase cDNA primer in ChIP assays. In the control cells, there was a moderate amount of H4 acetylation and no detectable H3 acetylation. As we recently reported [23], there was increased H3 acetylation without any change in overall H4 acetylation in the presence of T_3_. When cells were treated with cAMP, there was an increase in H4 acetylation without any measurable H3 acetylation. When cells were treated with T_3_ and cAMP, both H3 acetylation and increased H4 acetylation occurred. Taken together, these findings suggest that H3 acetylation occurs with T_3_ treatment whereas increased H4 acetylation occurs with cAMP treatment.

**Figure 3:**
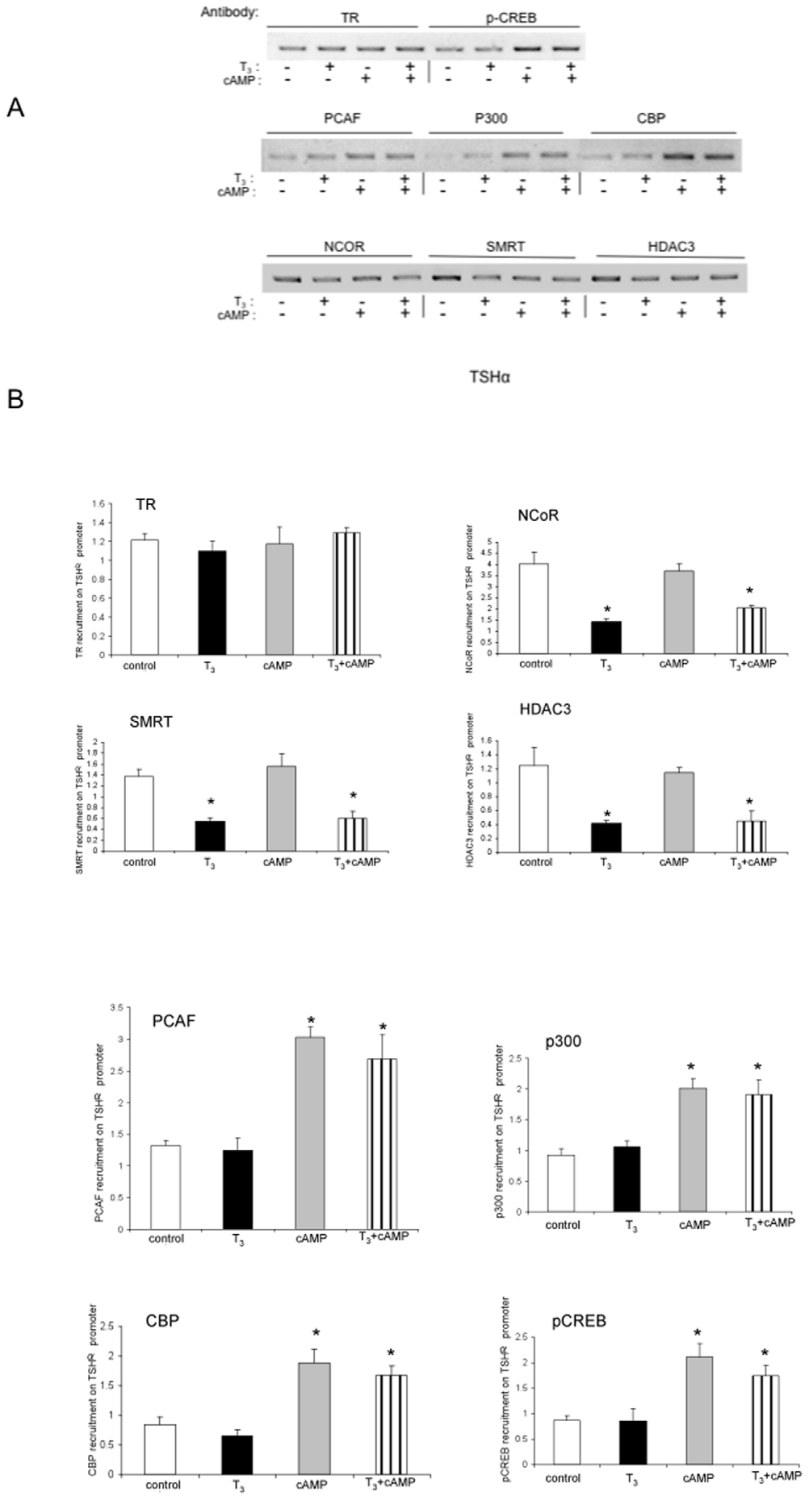
Cofactor binding to TSHα promoter with cAMP and T_3_ treatment. Corepressor complex and coactivator binding to TSHα promoter in the absence and presence of T3 cAMP. α-23 cells were treated for one hour with-/+ 0.1 µM T3 and -/+ 1 mM cAMP and harvested as in Figure 1C and D. ChIP assays were performed similar to Figure 1C. Antibodies used in ChIP assay are indicated in Figure and described in Materials and Methods. A) T_3_ and cAMP regulation of cofactor binding to the TSHα promoter. ChIP assay was performed as described in Materials and Methods using antibodies against indicated co-factors. Similar findings were observed in two other experiments. Note: “- ” indicates no hormone or cAMP treatment. B) Quantitative RT-PCR analyses of PCR products from three separate ChIP experiments using same antibodies as in Figure 3A (n = 3). The statistical analysis was performed utilizing ANOVA. Values are expressed as the mean ± SD. *, *P*<0.05 difference from control.

In order to examine the co-factors that may be involved in these changes in histone acetylation, we performed ChIP assays to examine co-factors bound to the TSHα promoter under all four conditions (Figure 3A, B). As reported previously, T_3_ caused decreased NCoR, SMRT, and HDAC3 binding to the promoter but did not affect TR binding [23]. cAMP alone did not change binding of these co-factors; however, concomitant addition of T_3_ decreased their binding. These findings suggest that loss of the NCoR/SMRT/HDAC3 complex binding is likely a major contributor to the negative regulation of TSHα gene by T_3_ even in the presence of cAMP. In contrast, pCREB, CBP, p300, and PCAF all increased their binding to the TSHα promoter after cAMP treatment; however, their binding to the TSHα promoter was unaffected by T_3_. These latter findings suggest that recruitment of this HAT complex likely causes the increased H4 acetylation on the TSHα promoter observed with cAMP treatment.

**Figure 4:**
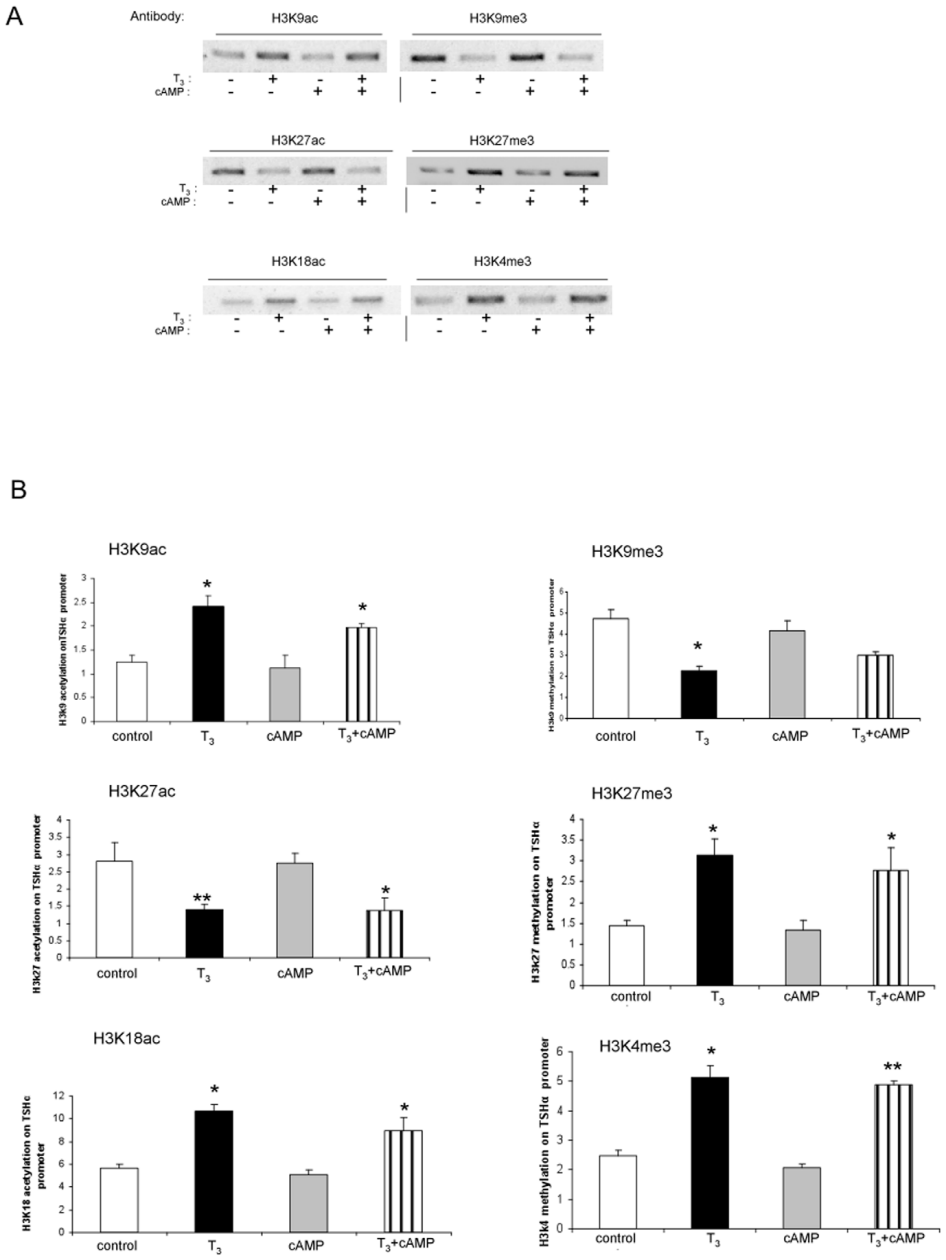
Specific histone H3 modifications on TSHα promoter with cAMP and T_3_ treatment. α-23 cells were treated for one hour with no hormone/cAMP, 0.1 µM T_3_, 1mM cAMP or both before harvest and ChIP assay. A) Specific histone H3 modifications. Shown are bands from gel electrophoresis of PCR products from ChIP assay using indicated anti-acetylated and anti-methylated H3 antibodies as indicated in the Figure. Similar findings were observed in two other experiments. B) Quantitative RT-PCR analyses of PCR products from three separate ChIP experiments using same antibodies as in Figure 4A (n = 3). Statistical analyses performed as in Figure 1. *, p<0.05.

We next examined acetylation of specific H3 sites under the same four conditions (Figure 4A, B). T_3_ increased H3K9 and H3K18 acetylation and decreased H3K27 acetylation. These findings suggest that although T_3_ induced an overall increase in H3 acetylation, it also decreased histone acetylation at H3K27. We then examined methylation at specific H3 sites. H3K9 and H3K27 showed reciprocal changes when compared to acetylation at these sites as T_3_ decreased H3K9, and increased H3K27 trimethylation. H3K4 methylation, which usually is associated with increased transcriptional activity [25], also was increased by T_3_. cAMP did not alter modifications on any of the foregoing H3 sites in the absence or presence of T_3_. Taken together, these data further confirm our previous findings [23] that T_3_ preferentially modulates H3 acetylation and methylation of the hTSHα promoter in our system.

**Figure 5:**
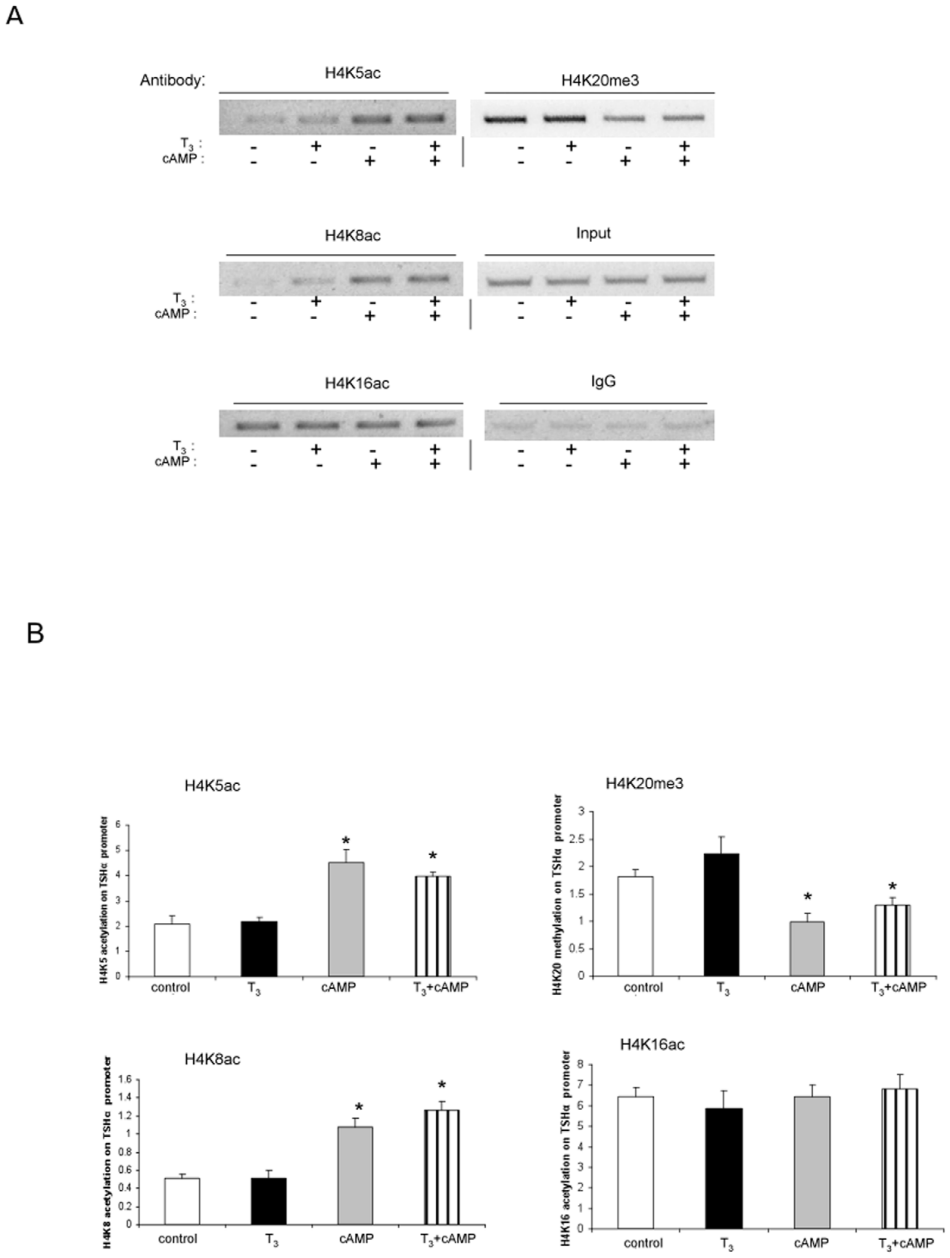
Specific histone H4 modifications on TSHα promoter with cAMP and T_3_ treatment. α-23 cells were treated for one hour with no hormone/cAMP, 0.1 µM T_3_, 1 mM cAMP or both before harvest and ChIP assay. A) Specific histone H4 modifications. Shown are bands from gel electrophoresis of PCR products from ChIP assay using indicated anti-acetylated and anti-methylated H4 antibodies as indicated in the Figure. Similar findings were observed in two other experiments. B) Quantitative RT-PCR analyses of PCR products from three separate ChIP experiments using same antibodies as in Figure 5A (n = 3). Statistical analyses performed as in Figure 1. *, p<0.05.

We then examined histone modifications at specific H4 sites (Figure 5A, B) under the same conditions. T_3_ did not change the acetylation or methylation on any of the H4 sites examined. In contrast, cAMP increased H4K5 and H4K8 acetylation without affecting H4K16 acetylation. It also decreased H4K20 trimethylation, which has been associated with transcriptional repression [25], [26]. Given the lack of any changes observed on H3, these findings suggest that cAMP caused both site-specific and histone-specific modifications on H4. H3S10 phosphorylation also has been associated with transcriptional activation [10]; however, we did not observe any phosphorylation changes at this site with either T_3_ or cAMP treatment (data not shown).

**Figure 6:**
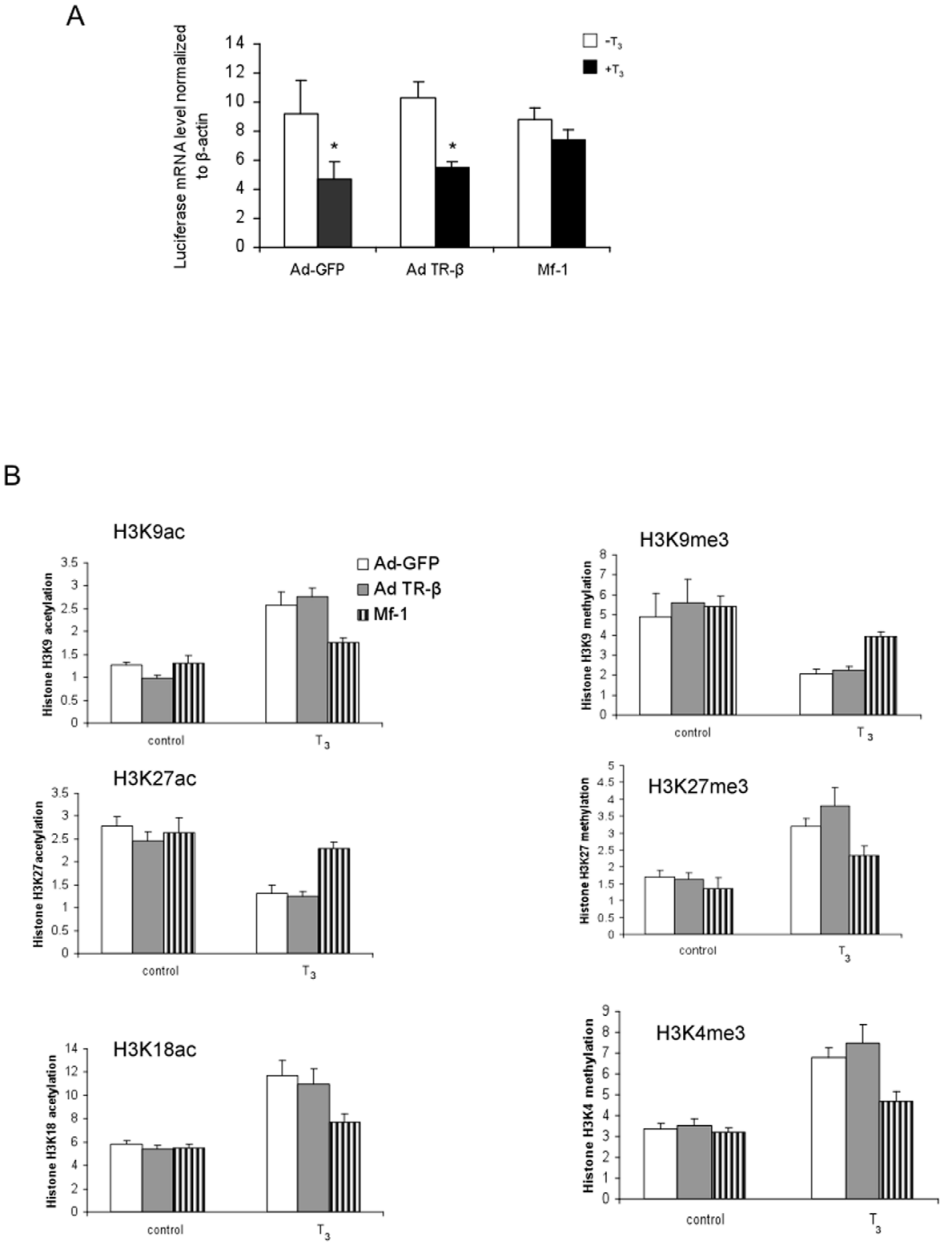
T_3_ effects on TSHα promoter activity and histone H3 modifications in α-23 cells overexpressed with wild-type TRβ and dominant negative mutant Mf-1. A) TSHα promoter activity in α-23 cells transformed with adenovirus green fluorescent protein (Ad-GFP), adenovirus TRβ (Ad-TRβ) and adenovirus Mf-1. α-23 cells were plated in 12 well plates and cultured in charcoal-stripped 10% FBS-DMEM for three days. Cells were transformed with adenovirus TRβ or Mf-1 on the fourth day. On the fifth day, cells were treated for 1 hr -/+ 0.1 µM T_3_ before harvest and measurement of luciferase mRNA expression by quantitative RT-PCR. B) Histone H3 modifications on TSHα promoter in α-23 cells transformed with adenovirus expressing GRP, TRβ, or Mf-1. α-23 cells transformed with adenovirus GFP, TRβ, or Mf-1 were treated for 1 hour with −/+ 0.1 µM T_3_ before harvest and ChIP assay. Shown are quantitative RT-PCR analyses of PCR products from using antibodies directed against specific H3 modifications as indicated in the Figure (n = 3 experiments). Statistical analyses performed as in Figure 1. **, p<0.05; *, p<0.01 from untreated and treated controls, respectively using ANOVA analyses.

Resistance to thyroid hormone (RTH) is a clinical syndrome in which most affected patients have a mutant TRβ allele that encodes a mutant receptor which has decreased binding affinity for T_3_ leading to decreased transcriptional activity [27]. The mutant receptor can still bind to TREs of target genes resulting in dominant negative activity on the transcription by the wild-type TRs, most likely due to competition with the latter for binding to TREs. To better understand the mechanism of the dominant negative activity on the negative regulation of TSHα gene expression, we examined the effects of a mutant TR (Mf-1) from a RTH patient [28], on histone modifications of the TSHα promoter in the absence or presence of T_3_ using adenovirus-GFP control as well as adenovirus encoding wild-type TRβ or Mf-1 in α-23 cells (Figure 6A). In cells transformed with adenovirus control and adenovirus TRβ, luciferase mRNA expression decreased to similar levels 1 hour after treatment with T_3_. In contrast, luciferase mRNA expression remained essentially unchanged after T_3_ treatment in cells transformed with adenovirus Mf-1. The protein expression of wild-type TRβ and Mf-1 in transformed cells was confirmed by Western blotting (data not shown). We next examined H3 modifications that were induced by T_3_ (Figure 6B), Mf-1 partially blocked the increases in H3K9 and H3K18 acetylation, and the decrease in H3K27 acetylation. It also blocked the T_3_-induced decrease in H3K9 methylation and increases in H3K27 and H3K4 methylation. These findings suggest that Mf-1 exerts its dominant negative activity by blocking all of the observed T_3_-induced H3 modifications of the TSHα promoter.

## Discussion

**Figure 7:**
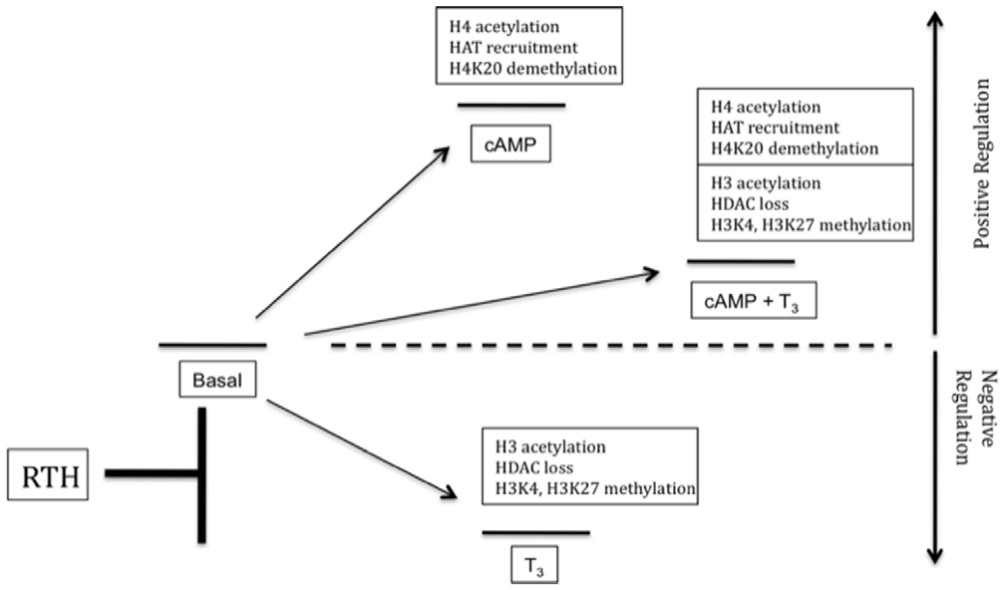
Diagram of effects of cAMP and T_3_ on transcription of TSHα gene. Upper boxes denote histone and histone-modifying enzyme changes associated with each treatment condition and transcriptional state. In RTH, histone modifications associated with negative regulation are partially blocked.

Our studies examined the histone modifications involved in negative and positive regulation of TSHα by T_3_ and cAMP, respectively. Several conclusions can be drawn from our current and previous findings [23]. First, histone acetylation can occur during both negative and positive regulation of the same target gene, as the chromatin changes induced by T_3_ and cAMP lead to opposite effects on transcriptional activity and recruitment of RNA pol II [23] (Figure 3), respectively, on the TSHα promoter. Second, NCoR/HDAC3 complex appears to determine the basal level of histone acetylation and transcription of the TSHα gene in the absence of any treatment (Figures 3 and 7). In the presence of T_3_, this complex dissociates from the promoter resulting in increased H3 acetylation whereas in the presence of cAMP, it remains bound to the TSHα promoter. pCREB then recruits co-activators with HAT activity, leading to both increased H4 acetylation and transcriptional activity. When cells are treated with both T_3_ and cAMP, NCoR/HDAC3 complex dissociates while co-activators remain bound to the promoter leading to an intermediate level of transcriptional activity (Figures 1, 3, and 7). These findings show that pCREB binding to CREs and the resultant transcriptional activation is independent from TR complex binding to TREs even though T_3_ can oppose cAMP transactivation as previously reported [29]. Third, although CBP/p300 and PCAF (Figures 3) are not involved in the negative regulation of TSHα gene, it is possible that there may be another co-factor mediating the H3 acetylation involved in negative regulation. Additionally, this putative co-factor would be expected to preferentially induce H3 acetylation on the TSHα promoter. Previously, we showed that the increased histone acetylation by T_3_ does not significantly involve SRCs, p300, CBP or RIP140 [23], lending further support for the possibility that another co-factor with histone acetyltransferase activity (which does not recruit CBP, p300, or PCAF) may participate in the negative regulation of TSHα gene expression.

Our current and previous findings also show that an overall increase in acetylation of H3 leads to negative regulation of the TSHα promoter [23] whereas an increase in acetylation of H4 leads to transcriptional activation. However, when there is concurrent H3 and H4 acetylation, as in the presence of T_3_ and cAMP, negative regulation still occurs albeit at higher transcriptional levels. Thus, when both signals act on the TSHα gene, H4 acetylation does not prevent H3 modifications and their transcriptional consequences from occurring (*i.e*., negative regulation) (Figures 2 and 7). Interestingly, an increase in acetylation does not occur at all H3 acetylation sites. T_3_ causes an increase in acetylation of H3K9 and H3K18 but does not affect acetylation of H3K14 (Figure 3). Furthermore, in contrast to H3K9 and H3K18, H3K27 acetylation is decreased by T_3_. It is not known how H3K27 is de-acetylated after T_3_ treatment. It is possible that another histone deacetylase can be recruited with T_3_ treatment that acts preferentially at this site. It also is possible that the release of HDAC3 from the promoter after T_3_ treatment may enable it to act on H3K27 and reduce acetylation at that site. Of note, the anti-H3 antibody used in Figure 2 does not detect H3K27 acetylation (Edward Lusby, Millipore Biosciences Division, personal communication). Nonetheless, our findings raise the intriguing possibility that net H3 acetylation might be increased after T_3_ treatment while acetylation at specific sites still can be decreased. H3K27 acetylation is associated with transcriptional activation [25], [26] so its deacetylation combined with increased H3K27 trimethylation would be expected to lead to decreased transcriptional activity. Thus, it is not known whether the net increase in histone H3 acetylation and/or a dominant effect by the deacetylation/methylation of H3K27 plays a major role in the negative regulation of the TSHα gene. Our data also showed that acetylation of H4K5 and H4K8 participate in H4 acetylation by cAMP. These sites previously have been reported to be associated with CBP/p300/pCAF HAT activity [25], [26].

We observed changes in methylation on different histones of the TSHα promoter by T_3_ and cAMP. In the presence of T_3_, there was a decrease in H3K9, and an increase in H3K27, trimethylation. These methylations were reciprocal to the histone acetylation changes at these sites. Of note, H3K27 trimethylation previously has been associated with transcriptional repression [25], [26]. Additionally, H3K4 trimethylation increased in response to T_3_. H3K4 trimethylation is generally associated with transcriptional activation [25], [26]; however, ligand-induced H3K4 demethylation has been reported for nuclear hormone receptors, including TRs [9]–[12] and associated with recruitment of the demethylase, LSD-1. In this connection, it recently has been demonstrated that approximately 10% of target genes containing H3K4 trimethylation also had H3K27 trimethylation, and most bivalent genes had decreased transcription compared to those with H3K4 trimethylation alone [30]. As was the case for histone H3 acetylation, cAMP did not affect the trimethylation of these H3 sites either in the absence or presence of T_3_. In the presence of cAMP, H4K20 trimethylation decreased. Methylation at this site has been reported previously to be associated with transcriptional repression [25], [26] so concomitant demethylation of H4K20 and acetylation of H4K4 and H4K8 may contribute to the transcriptional activation of TSHα gene by cAMP.

While negative and positive regulation of TSHα gene expression by T_3_ and cAMP are due to changes localized to specific histones, this is not necessarily the case for other target genes. We have observed both H3 and H4 acetylations in T_3_-mediated transcriptional activation of several endogenous target genes in α-23 cells such as growth hormone, cyp7, PEPCK, and SERRCA [23]; Wang and Yen, unpublished results). In particular, we have observed H3K9 and H4K16 acetylation in these target genes. Moreover, the acetylation of certain sites involved in the negative regulation of TSHα (*e.g.,* H3K9), also commonly occurs in the positive regulation of these and other target genes by T_3_ [10]. Our findings thus suggest that individual target genes may employ unique sets of histone marks to mediate either positive or negative regulation.

In the clinical syndrome of RTH, patients typically have elevated circulating thyroid hormone levels (T_3_ and T_4_) with inappropriately normal or elevated serum TSH levels. The latter occurs due to the lack of negative feedback control by thyroid hormones on the expression of TSHα and TSHβ subunits in pituitary thyrotropes. This dysregulation in TSH secretion occurs at the transcriptional level and is frequently due to expression of a mutant TRβ which has dominant negative activity on the transcriptional activity of wild-type TRs. In our studies, α-23 cells transformed with adenovirus that expressed the mutant TRβ from a patient with RTH, Mf-1, showed diminished negative regulation of TSHα gene expression in the presence of T_3_. Mf-1 also significantly blocked the increases in histone acetylation at H3K9 and H3K18 and the decrease in histone acetylation at H3K27 (Figure 6) as well as overall histone H3 acetylation (Wang and Yen, unpublished results). Additionally, Mf-1 partially blocked the decrease in H3K9 methylation and increases in H3K4 and H3K27 methylation. Thus, Mf-1 blocked each of the previously characterized H3 modifications induced by T_3_. It is likely that some or all of these alterations in T_3_-induced changes in chromatin structure may account for Mf-1's dominant negative activity on TSHα transcription and the loss of negative feedback control of TSH by T_3_ seen in RTH patients (Figures 6 and 7). With regard to positive regulation, we also have observed that Mf-1 is able to block key histone modifications (*e.g.,* H3K9Ac and H3K4Me3) in several positively-regulated endogenous target genes in α-23 cells (Wang and Yen, unpublished results). These findings suggest that disruption of T_3_-dependent histone modifications by dominant negative mutant TRs may account for the abnormalities in target gene expression observed in RTH patients.

In summary, we have shown that negative and positive regulation of the TSHα gene involves histone type- and histone site-specific modifications. Moreover, these histone modifications were different for positive regulation by cAMP than for negative regulation by T_3_. Thus, it appears that these independent and non-overlapping modifications enable T_3_ to negatively regulate TSHα gene expression and cAMP to mediate an increase in basal transcription level. TRH stimulates intracellular cAMP concentration and activates TSHα gene expression via pCREB binding to the CREs located in the proximal promoter region [24], [31]. Thus, the persistent negative regulation of TSHα gene in α-23 cells in the face of cAMP-mediated stimulation may explain the clinical observation of a rapid decrease in TSH levels after acute T_3_ administration in hypothyroid patients [32]. Moreover, our findings with Mf-1 suggest that the mechanism for inappropriate TSH secretion in patients with RTH is likely due to blockade of key histone modifications normally induced by thyroid hormone on the TSHα subunit promoter. Our studies highlight some of the key similarities and differences in co-factor recruitment and histone modification that occur during the negative and positive regulation of a target gene. They also demonstrate how opposing hormonal and intracellular signals can be integrated via histone modifications that determine chromatin structure and transcriptional activity of the promoter.

Last, our studies demonstrate some of the difficulties in interpreting and predicting transcriptional responses based upon individual histone modifications; and thus, suggest that an examination of a set of modifications on a given target gene, as performed herein, will be more informative. It will be interesting and worthwhile to see whether similar epigenetic changes for positive and negative regulation occur on a genome-wide basis in other target genes regulated by TRs, other nuclear hormone receptors, and transcription factors involved in other signaling pathways. Additionally, it is expected that disruption of histone modifications of target genes by aberrant hormonal and intracellular signals will be involved in the pathogenesis of other endocrine and metabolic diseases.

## Materials and Methods

### Reagents

Dibutyryl cyclic AMP (dbcAMP) and triodothyronine were purchased from Sigma–Aldrich, St. Louis, MO. Adenovirus expressing wild-type TRβ and dominant negative mutant TRβ (Mf-1) have been described previously [28], [33]. α-23 cells were generated from rat pituitary GH3 cells permanently-transfected with human TSHα promoter spanning −846 to +1 kindly provided by Dr. Larry Jameson (Northwestern University) [19], [23].

### Antibodies

Antibody against acetyl-Histone3 and 4(AcH3, AcH4) were purchased from Upstate Biotechnology/Millipore (06-599, 06-866). Other histone acetylation and methylation antibodies were purchased from Abcam, Cambridge, UK and include antibodies against: histone acetyl H3K9 (ab4441), acetyl H3K27 (ab4729), acetyl H3K18 (ab1191), tri-methyl H3K4 (ab8580), tri-methyl H3K27 (ab6002), tri-methyl H3K9 (ab8898), tri-methyl H4K20 (ab9053), acetyl H4K5 (ab51997), acetyl H4K8 (ab15823), and acetyl H4K16 (ab23352). Antibodies against NCoR(C-20), SMRT(C-19), HDAC3(H-99), PCAF (E-8), p300 (C-20),CBP (A-22), TR(C-1) were obtained from Santa Cruz Biotechnology, Santa Cruz, CA. Anti-phospho-histone H3 (Ser10) was purchased from Millipore, Billerica, MA. Anti-CREB antibody was purchased from Cell Signaling Technology, Danvers, MA.

### Cell culture, transient transfection, and reporter analyses

Monolayer cultures of clone α- 23 cells were grown in Dulbecco's modified Eagle's medium (LifeTechnologies, Carlsbad, CA) supplemented with 10% heatedinactivated fetal calf serum (Biofluids, Rockville,MD) and maintained in 5% CO_2_ atmosphere at 37°C. Cells were transfected with 300 ng DNA/well in 12-well plates with Lipofectamine 2000 (Invitrogen, Carlsbad, CA) according to the manufacturer's instructions. Cell culture media was changed 6 hours after transfection to antibiotic-free DMEM plus 10% charcoal-dextran treated fetal bovine serum. After 48 hours, 10^−7^ M T_3_ or 1 mM dbcAMP in fresh media were added to the cells, and cells were harvested, lysed and assayed for reporter gene activity the next day (16 hours later) using dual luciferase assay reagents according to the manufacturer's instructions (Promega, Madison, WI).

### Adenoviral infection

293-HEK cells were transformed with adenoviruses expressing wild-type TRβ or dominant negative mutant Mf-1 [34]. Cell lysates were obtained after 5 days, and then were added to 293 cells again. When most of the cells were killed by the adenovirus infection and had detached, cell lysates were obtained again. This process was repeated three times. Control adenovirus expressing green fluorescent protein (Ad-GFP) was prepared in the same manner. The adenovirus preparations then were purified and concentrated by CsCl_2_ gradient ultracentrifugation two times. After determining viral titres, α-23 cells (400,000 per 35-mm well) were plated in culture media for two days, then washed twice with PBS, and infected with adenovirus at a concentration of 50 viral particles per cell in 0.5 ml of culture medium. One hour later, 1.5 ml of fresh culture medium was added to the cells which then were allowed to grow for 24 hours before treatment with -/+ 10^−7^ M T_3_ for one hour. Cells then were harvested, their RNA extracted, and quantitative RT-PCR performed.

### mRNA measurement quantitative RT-PCR

Total RNA was isolated from α-23 cells using TRIzol reagent (Invitrogen, Carlsbad, CA). cDNAs were synthesized using random primers and MultiScribeTM reverse transcriptase (Applied Biosystems, Foster, CA). qRT-PCR was performed using SYBR Green Master Mix (Applied Biosystems, Foster, CA) and Applied Biosystems 7300 sequence detector. The following qRT-PCR oligonucleotide primers sets were used: Luciferase 5′-CCAGGGATTTCAGTCGATGT-3′ and the reverse primer 5′-AATCTGACGCAGGCAGTTCT-3′; rat beta-actin, forward, 5′-CGCCGTTCCGAAATTGC 3′, reverse 5′- GCCGCCGGGTTTTATAGG 3′. 0.5 µM primers were used in the PCRs [23].

### Chromatin immunoprecipitation (ChIP) assay

ChIP assay was performed as previously described [7]. Briefly, α-23 cells were grown to 90% confluence in phenol red-free Dulbecco's modified Eagle medium (DMEM) supplemented with 10% charcoal DEXTRAN-stripped FBS for at least 3 days. After addition of 10^−7^ M T_3_ or 1 mM dbcAMP for one hour, ChIP assays were performed according to manufacturer's protocol (Upstate Biotechnology, Lake Placid, NY) with some minor modifications. Briefly, chromatin was cross-linked in 1% formaldehyde in phosphate buffered saline (PBS), and nuclei were extracted. Chromatin was sonicated to yield 500- to 1,000-bp DNA fragments and the supernatant containing precleared chromatin was then incubated at 0°C overnight with different antibodies or rabbit IgG control. After reverse cross-linking by heating the samples at 65°C overnight, and treating with Proteinase K. DNA was purified using Qiagen PCR Purification Kit per manufacturer's instructions. PCR was performed to visualize the enriched DNA fragments.using primers which amplify the promoter region of TSHα in pGL2/TSHα plasmid and part of the luciferase cDNA (Figure 1) [22]. Primers used were: 5′CAGGATGTTATGTGTATGGCTC3′ for the TSHα promoter and 5′CTTTATGTTTTTGGCGTCTTC3′ for the luciferase gene. The upstream primer started at -324 bp and the downstream primer started at +23 bp from transcriptional start site of luciferase gene. Conventional PCR signals were stained with ethidium bromide in 2% agarose gels.

Quantitative analyses of DNA products obtained from ChIP assay were performed by RT-PCR with primers specific for the human TSHα promoter [22]. RT-PCRs conducted on DNA derived from input chromatin templates served as positive controls whereas reactions conducted on IgG-precipitated templates served as negative controls. In addition, we performed RT-PCRs using primers to human TSHα promoter sequences to which TR and other co-factors do not bind as well as to internal luciferase cDNA sequences. The RT-PCR signal was barely detectable for these controls. The signal for these samples and IgG-precipitated templates was negligible on gels (Figure 5 and data not shown).

Relative amounts of immunoprecipitated co-factors or modified histones (mean ± S.D.) in ChIP assays are shown on the Y axes of Figures 2D,3B, 4B, 5B, and 6B and determined from quantitative qRT-PCR data using SYBR Green Master Mix (Applied Biosystems, Foster, CA) and Applied Biosystems 7300 sequence detector. Fluorescence from IgG precipitated samples was subtracted from fluorescence from samples. Threshold cycles (C_t_) then were determined for ChIP samples and the input DNA, and the relative amount of immunoprecipitated DNA (% ChIP signal per input DNA) was calculated as 100×2 ^ΔCt^.

### Statistical Analysis

The statistical analysis was performed utilizing an analysis of variance (ANOVA). Values are expressed as the mean ± standard deviation of the mean (SD). The significance of differences between the mean values was evaluated using the paired Student's t-test.
